# Designing, implementation and evaluation of story reading: a solution to increase general empathy in medical students

**DOI:** 10.1186/s12909-024-05384-4

**Published:** 2024-04-30

**Authors:** Masoumeh Mahmoudi, Ali Asghar Ghorbani, Mehdi Pourasghar, Azita Balaghafari, Jamshid Yazdani Charati, Nassim Ghahrani, Farzaneh Amini

**Affiliations:** 1 Department of General Education, School of Paramedical Sciences, Mazandaran University of Medical Sciences, Sari, Iran; 2 School of Paramedical Sciences, Mazandaran University of Medical Sciences, Sari, Iran; 3 Psychiatry and Behavioral Sciences Research Center, Addiction Institute, Mazandaran University of Medical Sciences, Sari, Iran; 4 Department of Health Information Technology, School of Paramedical Sciences, Mazandaran University of Medical Sciences, Sari, Iran; 5 Department of Biostatistics and Epidemiology, School of Health, Health Sciences Research Center, Mazandaran University of Medical Sciences, Sari, Iran; 6 Mazandaran University of Medical Sciences, Sari, Iran; 7 Department of Biostatistics and Epidemiology, Student Research Committee, School of Health, Mazandaran University of Medical Sciences, Sari, Iran

**Keywords:** Story reading, General empathy, Medical students, Interventional education

## Abstract

**Background:**

Communication and mutual understanding among healthcare providers is a significant concern within the healthcare system, and enhancing empathy is one way to foster effective communication and mutual understanding. The aim of this research is to evaluate and compare the impact of story reading on the level of empathy in medical students at Mazandaran University of Medical Sciences.

**Methods:**

The study employed an intervention educational design (a quasi-experimental), with a convenience sample of 51 medical students selected as the statistical population. The process of story reading was conducted over six two-hour virtual sessions in the students' classroom, spanning six weeks. Selected stories were discussed in an online virtual class under the supervision of an instructor, focusing on story elements. To assess students' empathy in this educational program, the Davis General Empathy Questionnaire was administered before each of the six sessions, after, and one week later at the end of the course. Statistical analysis of the collected data was performed using repeated measures analysis of variance and Bonferroni's post hoc test through SPSS version 28 software, with a significance level set at 0.05.

**Results:**

The findings revealed that 27 participants (58.7%) were female students, with the remaining being male students, having an average age of 19.5 ± 0.86 years. The level of general empathy among the students significantly increased after the intervention compared to before the intervention (*P*<0.001). Furthermore, the analysis of variance with repeated measures indicated a significant effect of the story reading program on enhancing empathy in terms of emotional and cognitive transfer among students in the intervention group (*P*<0.001).

**Conclusions:**

The research findings suggest that the story reading program effectively enhances the overall sense of empathy among medical students at the University of Medical Sciences. Therefore, implementing this method in universities, higher education centers, libraries, and psychology centers for teaching empathy can be valuable in fostering empathy skills and improving healthcare.

**Supplementary Information:**

The online version contains supplementary material available at 10.1186/s12909-024-05384-4.

## Introduction

In the realm of medicine, even with remarkable technological progress, it remains imperative to maintain effective communication and personal interactions when interacting with and providing care for patients. Research outcomes underscore the importance of the doctor-patient relationship in comprehending and addressing the suffering caused by illnesses, thereby emphasizing the necessity to enhance this relationship [1]. Many researchers acknowledge the importance of empathy among healthcare professionals and the value of training in developing this skill [2, 3]. Furthermore, investigations into complaints related to doctors confirm that many of these grievances are not linked to the doctors' scientific skills or efficiency but rather arise from their communication approach with patients [4]. In simple terms, it can be said that communication errors are the primary cause of most medical complaints and violations. While information and communication technology (ICT) have made obtaining health and medical information easier, medical technology cannot validate the doctor-patient relationship, which is built upon their mutual attitudes towards one another. As a result, a therapist's behavior may vary depending on their perception of and attitude towards their patient since people's behavior is influenced by their orientation towards others and their perceptions.

Carl Rogers, a renowned humanist in the early 20th century, believed that empathy was a crucial and effective process for facilitating psychological changes within the doctor-patient relationship [5]. Furthermore, doctor-patient empathy holds a significant ethical importance within the medical community [6], which has led to discussions on this topic during medical ethics congresses. The need for well-designed educational plans to enhance empathetic abilities is highlighted by research that indicates a lack of satisfaction in this area [7]. Empathy training can strengthen this moral virtue and improve the mental and spiritual well-being of patients by incorporating it into the medical curriculum [8]. Empathy skills will help healthcare providers better understand and address their patients’ emotional needs, thereby fostering more positive therapeutic outcomes.

We are narrative creatures and we grow by telling stories and listening to stories [9]. Many believe that storytelling has many benefits and as an educational tool can improve the ability to retain words, improve their vocabulary, encourage children to learn English. , increasing moral value in them and providing cheap media in teaching rich language experience and increasing students' interest in reading and improving listening and writing abilities [10]. Also some narration and storytelling are considered useful for treatment, intervention and as a tool and technique for collecting qualitative data about treatment processes [11]. There is a view that how to read a story and get to know the structure of a story text in literature has similarities with narrative medicine. In fact, just as literature deals with the sufferings and problems of humans, narrative medicine mainly deals with the sufferings and problems of patients, patients' families or health care doctors. Therefore, the narrative reading techniques used in the literature study can be used in the study of narrative medicine to better understand the suffering of patients and thus help medical students and health care professionals to communicate with patients. And this can be effective in creating empathy [12]. To apply the narrative approach in teaching, we must answer the key questions that are necessary to understand fiction; Questions like who, what, where, when and why? What is the storyline? How will it begin, develop and end? [13]. Indeed, the field of narrative medicine has provided a useful way to open up our understanding of clinical reasoning, as we realize that the task of arriving at a diagnosis and treatment plan is largely narrative in nature [14].

In addition, many researchers in the field of medical humanities [15], medical professionalism [16] and the study of literary fiction in the creation and development of clinical abilities for compassionate and professional interaction with the patient has been highlighted by the importance and significance of phenomenological research and the lived experience of illness [17]. They have used experimental methods such as FMRI to track the effect of reading a story text on brain activities and its positive effect [18]. Since reading literary fiction is a cheap and effective way to develop empathy, story-reading and discussion sessions about the plot of tales has been held for years for hospital staff [19].

Of course, many of these studies are dedicated to reading a text or a part of a literary text and that too in one session, and the research community in them consists of people who have a tendency to read a story text, so it is possible that the influence of the text is due to the tendency and preparation of them to learn more empathy. Another important aspect is that while these studies highlight the impact of reading literary stories on readers, improving their communication skills and fostering empathy, none of them have been specifically designed to incorporate educational lessons centered on text analysis and teaching of story elements as this current study does. Although we have not discovered any evidence of a similar process being implemented in educational institutions nationwide, internal studies have suggested that reading stories can have an impact on reducing behavioral inconsistencies or enhancing learning abilities [20, 21].

Considering the researches that have raised the need for training and creating empathy among therapists (8, 40, 41 and 42), we decided to include reading the text of fiction and familiarizing with the elements of story structure in the curriculum of medical students. Our research has several features: using Persian stories, reading stories along with explaining and discussing their story elements, and placing these training sessions in the students' curriculum and implementing it in 6 two-hour training sessions.

These four theories (cognitive Learning theory, behaviorism Learning theory, Constructivism Learning and Connectivism Learning) can be effective in storytelling. According to the cognitive theory, internal and external factors can affect the learners, and in the behaviorist learning theory, the concept is emphasized that the behavior of the learners depends on how they interact with their environment. In constructivism learning theory, the learner designs its learning based on previous experiences, and Connectivism learning theory focuses on the concept that people learn and grow when creating relationships [10].

Therefore, it seems that storytelling and active exposure to story can be considered by each of these theories, and considering the relationship between the understanding of the text structure of fictional story and the story of patients and diseases, storytelling and knowing the elements and structure of the story can be effective in creating narrative skills in learners.

Currently, although the significance of empathy skills is acknowledged in courses such as medical ethics, the teaching of this skill is not officially sanctioned in medical courses. Given the impact of accurately understanding a story in cultivating empathy, it is worth considering the inclusion of indirect instruction on this skill in the Persian literature course. This course is a three-unit general course. Medical students are mandated to successfully complete three units of Persian literature as part of their curriculum. The course content has been authorized by the Ministry of Science, although the Ministry of Health's educational planning office has yet to designate an official curriculum for it. Despite being approved by the Ministry of Science with the aim of providing comprehensive education in the Persian language and developing proficient reading and writing skills, critics argue that the general Persian course falls short in adhering to the approved curriculum. They claim that the lecturers, disregarding the audience's needs, tend to design educational content based on their personal preferences and areas of expertise [22, 23]. In recent years, the importance of focusing on empathy skills among medical staff and the need for their training has consistently been emphasized in medical ethics meetings and conferences. Therefore, the authors of this article recognize the significance of enabling medical students to enhance their empathy skills. They also acknowledge the potential impact that reading literary stories can have on improving this skill. Consequently, they have decided to focus on teaching the art of accurately interpreting stories. Therefore, this study aims to evaluate and compare the impact of story reading on the level of empathy in medical students at Mazandaran University of Medical Sciences.

## Methods

We designed an intervention educational study (a quasi-experimental) with utilizing the ADDIE model, which comprises of five primary elements: Analysis, Design, Development, Implementation, and Evaluation. Out of the 51 medical students (Second year) enrolled at medical school of Mazandaran University of Medical Sciences in Sari, 46 students participated in this research study during the first semester of the academic year 2021. During the design phase, the researchers developed the stories, execution method, and test method. Before each session, the students read the selected stories specific to that session. Then, they were required to participate in an exam based on the content of the stories and the session. To assess the students' empathy levels (Design phase), we utilized the Davis empathy questionnaires prior to and following the sessions. Three subscales of the IRI (Interpersonal Reactivity Index) (Davis, 1980) were used to assess empathic concern (EC), perspective-taking (PT) and Fantasy Scale (FS). Consisted of seven items answered on a 5-point scale (describes me very well to does not describe me at all). Which each of subscales included seven items that were answered on a 5-point scale (very agree to very disagree).

The EC subscale measures the feelings of compassion and concern for others, also known as emotional empathy. For example, "I often become emotionally affected by witnessing events". The PT subscale measures the inclination to consider the psychological viewpoint of others, also known as cognitive empathy. For example, "I make an effort to pay attention to the opposing perspective of each person before engaging in a debate". Also, the FS subscale is a measure of an individual's tendency to identify with fictitious characters from books, films, or video games. (e.g., “When I am reading an interesting story or novel, I imagine how I would feel if the events in the story were happening to me”).

During the developmental phase, we gathered the stories using research methods that have established the impact of reading stories on empathy. For this study, we selected stories from prestigious literary festivals or renowned authors who have caught the attention of literary criticism theorists and were carefully chosen by literature experts. Additionally, this aspect is encompassed in the selection of stories that are deemed appropriate for teaching story elements during the assigned sessions. During the implementation phase, students who expressed disinterest in participating in the research were instructed to study the material covered in the in-person classes and respond to tailored questions in the final examination. Please note that the offline content lacks criticism and analysis of the story, and it consists of typical educational texts found in this course. Additionally, it includes ancient Persian texts that have been translated into modern Persian poetry and prose.

In this study, the reading of literary texts was conducted virtually and online in the classroom of medical students for a total of six 2-hour sessions over a period of six weeks. This arrangement was necessary due to the Covid-19 pandemic and the resulting limitations on in-person educational activities. The reason for allocating 2 hours of time for this training over 6 sessions is to equate it to 1 lesson unit. This unit will be included in the proposed subject of a 3-unit Persian literature course for medical students. In order to assess the students' empathy during this educational course, the Davis General Empathy Questionnaire was administered before each of the six sessions, after, and one week later at the end of the course.

Another important aspect is that during these sessions, the teacher offered explanations on story elements by referring to relevant specialized books. The stories selected for this study were based on the author's esteemed reputation and literary recognition, as supported by research in this field [24–29]. The stories were selected by the literature expert (MM) who taught the training sessions. These stories are known in Persian literature and have famous authors. As mentioned in the Additional file 1: Appendix 1, some of these stories have won awards from festivals, and the rest are famous writers of Persian literature, and have been selected from the book "Short Story in Iran" by Dr. Hossein Payandeh, a well-known Iranian critic, holder of a chair of literary criticism and Theorizing in Allameh Tabatabai University.

After every session, in order for the professor to make sure that the students' attention, listening and active presence in the class, a test was administered, which focused on the topics covered during that session. This was done in support of the measured empathy resulting from reading the story. Additionally, the level of student satisfaction with the educational material and the manner in which the sessions were conducted was gauged using an interview and self-expression by students. To assess the durability of empathy, students were required to complete the Davis General Empathy Questionnaire one week later at the end of the course.

## Results

We evaluated 46 students, including 27 (58.7%) female students and the rest male students with an average age of 19.5 ± 0.8. The highest age of the intervention subjects was 22 years and the youngest was 18 years. In this study, empathy was evaluated in three time periods before the intervention, after the intervention, and then 7 days after the end of the intervention, and the results listed below are related to the overall empathy scores (Table 1).

**Table 1:** Descriptive indices of overall empathy scores in three time periods before the intervention, after the intervention and then one week after the end of the intervention

TE	Mean	Std. Error	95% Confidence Interval	
			Lower Bound	Upper Bound
1	66.615	1.056	64.479	68.752
2	84.821	1.113	82.567	87.074
3	82.872	1.014	80.820	84.924

*TE* Total Empathy

To compare overall empathy, analysis of variance with repeated measurement was used because Mauchly's Test of Sphericity showed significant results (*P*<0.001), therefore Greenhouse- Geisser test was used and a significant difference was observed in three periods of overall empathy measurement (*P*<0.001).

For comparisons in three times of measuring empathy, the results listed in the following table were also obtained: the type of paired t-test and the significance level of 5%, which was considered to be 0.015 with Bonferroni's correction based on the three times of measurement (Table 2).

**Table 2:** Paired t-test for comparison of total empathy scores in three times of empathy measurement with Bonferroni correction

(I) TE	(J) TE	Mean Difference (I-J)	Std. Error	Sig.^b^	95% Confidence Interval for Difference^b^	
					Lower Bound	Upper Bound
1	2	-18.205^a^	.978	.000	-20.654	-15.757
	3	-16.256^a^	.952	.000	-18.641	-13.872
2	1	18.205^a^	.978	.000	15.757	20.654
	3	1.949^a^	.542	.003	.590	3.307
3	1	16.256^a^	.952	.000	13.872	18.641
	2	-1.949^a^	.542	.003	-3.307	-.590

Based on estimated marginal means

^a^The mean difference is significant at the .05 level

^b^Adjustment for multiple comparisons: Bonferroni

**Fig. 1:**
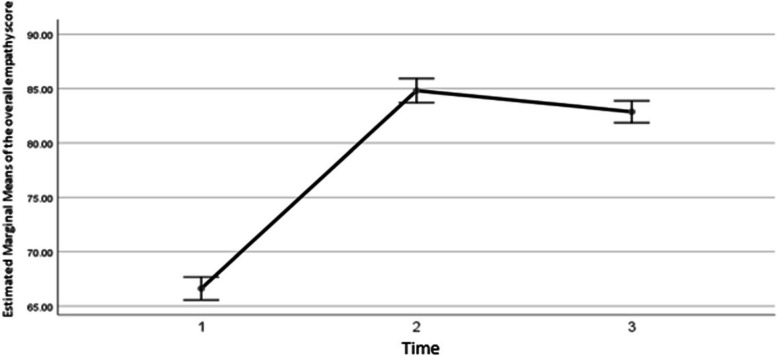
The changing trend of the overall average empathy score in three time periods

The results showed that the average empathy score increased by 18.2 ± 0.97 after the intervention (*P* < 0.001). And in the third time compared to the second time, it increased by 16.25 ± 0.95 (*P*<0.001). In the third time, it decreased by 1.94 ± 0.54 compared to the second time (*P*=0.003), whose graph is as follows (Fig. 1).

The results showed that the overall average score of empathy increased in the third time compared to the first time. In the first dimension of empathy (EC=empathic concern), the results were calculated as follows (Table 3).

**Table 3:** Descriptive indices of EC dimension, empathy scores in three time periods before the intervention, after the intervention and then one week after the end of the intervention

time	Mean	Std. Error	95% Confidence Interval	
			Lower Bound	Upper Bound
1	22.974	.516	21.929	24.019
2	28.897	.428	28.031	29.764
3	28.051	.420	27.200	28.902

The above results show the description of scores with a 95% confidence interval in the first dimension of empathy. To compare the first component of empathy, analysis of variance with repeated measurements was used, Mauchly's Test of Sphericity showed significant results (*P*<0.001), therefore Greenhouse-Geisser test was used and a significant difference was observed in the first three measurement periods of the first component of empathy (*P*<0.001). For comparisons, the results listed in the following table were obtained these three times by measuring the first dimension of empathy: the type of paired t-test and a significance level of 5%, which was considered to be 0.015 with Bonferroni's correction based on the three times of measurement (Table 4).

**Table 4:** Paired t-test for comparisons of EC dimension, empathy scores in three times of empathy measurement with Bonferroni correction

(I) EC	(J) EC	Mean Difference (I-J)	Std. Error	Sig.^b^	95% Confidence Interval for Difference	
					Lower Bound	Upper Bound
1	2	-5.923^a^	.497	.000	-7.169	-4.677
	3	-5.077^a^	.556	.000	-6.470	-3.683
2	1	5.923^a^	.497	.000	4.677	7.169
	3	.846^a^	.279	.013	.148	1.544
3	1	5.077^a^	.556	.000	3.683	6.470
	2	-.846^a^	.279	.013	-1.544	-.148

Based on estimated marginal means

^a^The mean difference is significant at the .05 level

^b^Adjustment for multiple comparisons: Bonferroni

**Fig. 2:**
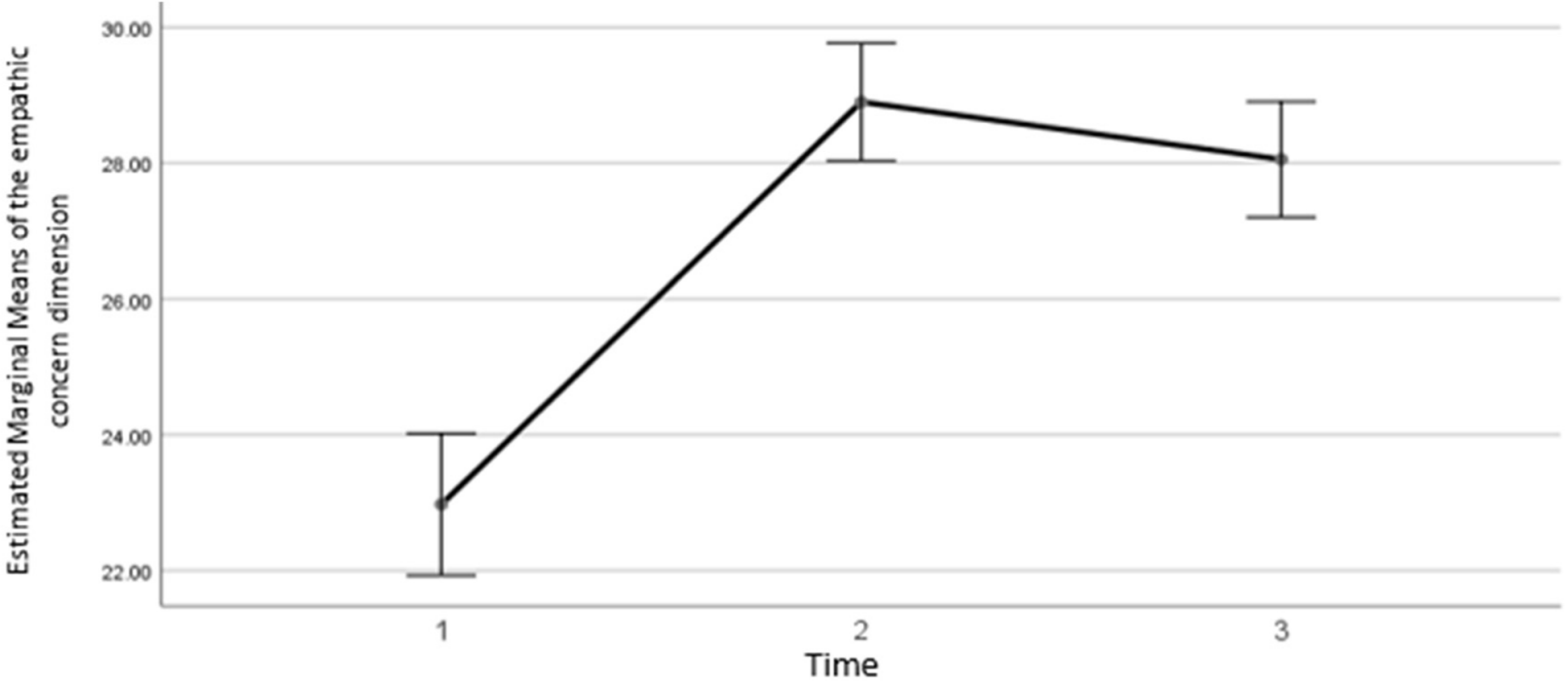
The changing trend of the average empathy score in the dimension of empathic concern in three time periods

The results showed that the average score of the first dimension of empathy increased by 5.92 ± 0.4997 after the intervention (*P* < 0.001). And in the third time compared to the second time, it increased by 5.07 ± 0.56 (*P*<0.001). Also, the third time, it decreased by 0.85 ± 0.28 compared to the second time (*P*=0.013) whose diagram is as follows (Fig. 2).

The results showed that the average score of empathy in the dimension of empathic concern increased in the third time compared to the first time. In the second dimension of empathy (PT=perspective-taking), the results were calculated as follows (Table 5).

**Table 5:** Descriptive indices of PT dimension, empathy scores in three time periods before the intervention, after the intervention and then one week after the end of the intervention

time	Mean	Std. Error	95% Confidence Interval	
			Lower Bound	Upper Bound
1	19.436	.447	18.532	20.340
2	25.795	.525	24.732	26.858
3	25.385	.561	24.248	26.521

The above results show the description of scores with a 95% confidence interval in the second dimension of empathy. To compare the second component of empathy, analysis of variance with repeated measurements was used, Mauchly's Test of Sphericity showed significant results (*P*<0.001), the Greenhouse-Geisser test was used and a significant difference was observed in the three measurement periods of the second component of empathy (*P*<0.001). For comparisons in these three times of measuring the second dimension of empathy, the results listed in the following table were also obtained: the type of paired t-test and the significance level of 5%, which was considered to be 0.015 with Bonferroni's correction according to the three times of measurement (Table 6).

**Table 6:** Descriptive indices of PT dimension, empathy scores in three time periods before the intervention, after the intervention and then one week after the end of the intervention

**Pairwise Comparisons**						
(I) PT	(J) PT	Mean Difference (I-J)	Std. Error	Sig.^b^	95% Confidence Interval for Difference^b^	
					Lower Bound	Upper Bound
1	2	-6.359^a^	.461	.000	-7.514	-5.204
	3	-5.949^a^	.486	.000	-7.166	-4.732
2	1	6.359^a^	.461	.000	5.204	7.514
	3	.410	.318	.613	-.385	1.206
3	1	5.949^a^	.486	.000	4.732	7.166
	2	-.410	.318	.613	-1.206	.385

Based on estimated marginal means

^a^The mean difference is significant at the .05 level

^b^Adjustment for multiple comparisons: Bonferroni

**Fig. 3:**
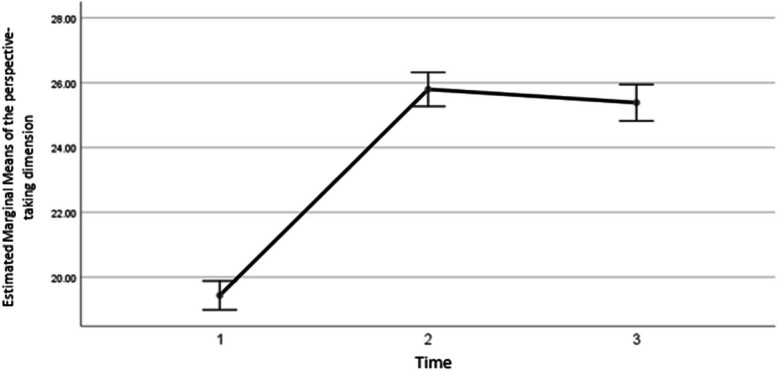
The changing trend of the average empathy score in the dimension of perspective-taking in three time periods

The results showed that the average score of the second dimension of empathy increased by 6.37 ± 0.46 after the intervention (*P*<0.001), and in the third time compared to the second time it increased by an average of 5.94±0.48 (*P*<0.001). Also, in the third time, no significant difference was observed compared to the second time (*P*=0.61), whose graph is as follows (Fig. 3).

The results showed that the average score of empathy in the dimension of e *perspective-taking* increased in the third time compared to the first time. In the third dimension of empathy (FS= Fantasy Scale), the results were calculated as follows (Table 7).

**Table 7:** Descriptive indices of FS dimension, empathy scores in three time periods before the intervention, after the intervention and then one week after the end of the intervention.

**Estimates**				
time	Mean	Std. Error	95% Confidence Interval	
			Lower Bound	Upper Bound
1	24.205	.427	23.340	25.070
2	30.128	.386	29.348	30.909
3	29.436	.392	28.643	30.229

The above results show the description of scores with a 95% confidence interval in the third dimension of empathy. To compare the third component of empathy, analysis of variance with repeated measurements was used, Mauchly's Test of Sphericity showed significant results (*P*<0.001), the Greenhouse-Geisser test was used and a significant difference was observed in the three measurement periods of the third component of empathy (*P*<0.001). For comparisons in these three times of measuring the third dimension of empathy, the results listed in the following table were also obtained: the type of paired t-test and the significance level of 5%, which was considered to be 0.015 with Bonferroni's correction according to the three times of measurement (Table 8).

**Table 8:** Descriptive indices of FS dimension, empathy scores in three time periods before the intervention, after the intervention and then one week after the end of the intervention

**Pairwise Comparisons**						
(I)FS	(J)FS	Mean Difference (I-J)	Std. Error	Sig.^b^	95% Confidence Interval for Difference^b^	
					Lower Bound	Upper Bound
1	2	-5.923^a^	.429	.000	-6.998	-4.849
	3	-5.231^a^	.473	.000	-6.414	-4.047
2	1	5.923^a^	.429	.000	4.849	6.998
	3	.692^a^	.252	.028	.060	1.324
3	1	5.231^a^	.473	.000	4.047	6.414
	2	-.692^a^	.252	.028	-1.324	-.060

Based on estimated marginal means

^a^The mean difference is significant at the .05 level

^b^Adjustment for multiple comparisons: Bonferroni

**Fig. 4:**
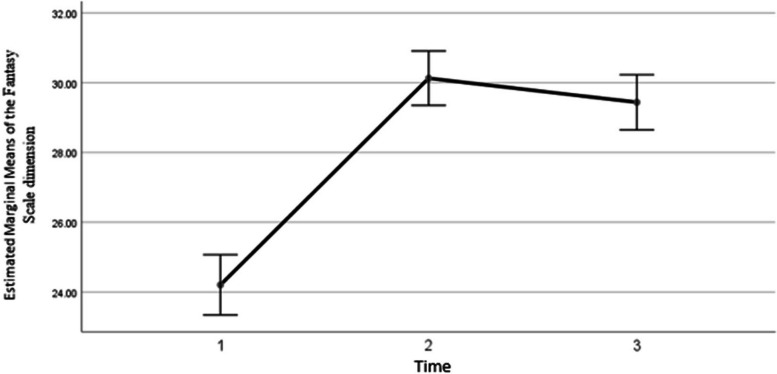
The changing trend of the average empathy score in the dimension of Fantasy Scale in three time periods

The results showed that the average score of the third dimension of empathy increased by 5.92 ± 0.43 after the intervention (*P*<0.001), and in the third time compared to the second time it increased by an average of 5.23±0.47 (P*<*0.001). Also, in the third time, no significant difference was observed compared to the second time (*P*=0.028), whose graph is as follows (Fig. 4). The results showed that the average score of empathy in the dimension of Fantasy Scale increased in the third time compared to the first time.

## Discussion

This study was conducted with the aim of investigating the effectiveness of reading stories in the Persian literature course on increasing empathy in medical students. The results of the present study indicated that reading stories had an effect on increasing empathy in the fields of emotional transfer (FS), cognitive and emotional empathy of students in the intervention group. In line with the current research, Maria et al.(2016) found that reading stories increases empathy among students [30]. Also, Matis et al. (2013), and Maja et al. (2013) showed the effectiveness of reading stories on empathy [31, 32].

Also, the findings of the present study indicated that the story reading program had an effect on increasing empathy in the emotional transfer dimension of medical students in the intervention group, and the effectiveness of the intervention was stable in the follow-up phase. In line with the findings of the present study, Mumper and Gerrig (2017) concluded that reading stories can improve empathy [33]. In the Netherlands (2013), during a university research, it was found that reading the story carefully and conveying it emotionally to the reader can increase their empathy [31]. The research of Djikic et al. (2013) also confirmed the same result [32].

Also, the findings showed that the story reading program had a significant effect on increasing empathy in the cognitive dimension of students in the intervention group. In line with this finding, Panro et al. (2016) concluded that reading stories can affect the cognitive dimension and improve empathy [34]. In the explanation of the mentioned findings, it can be said that since reading the story emphasizes the mental and cognitive abilities of humans, it can lead to the development of cognitive skills in people [35] and increase people’s aware of others cognitive, emotional and sense states [36]. The research of Kidd et al. (2016) also showed that reading stories in the long term can increase our understanding of the cognitive and emotional states of others [37]. Also, the findings showed that reading stories had a significant impact on emotional empathy. In line with these findings, Koopman et al. and Dickij et al. also reached these results [32, 38]. This finding is due to the fact that empathy is an important concept in the field of psychology and interpersonal communication. As a personal characteristic and also as an ability, it makes a person show desirable emotional reactions by perceiving the emotional reactions of others. In order to establish empathetic relationships, a person must be able to put himself in the place of others. Empathetic people are kind and caring towards others. They worry about others when they get hurt, on the other hand, their sensitivity to the behavior of others and trying to understand the nature of the behavior of the people around them makes them feel close and empathetic with the person to whom an incident has happened [39, 40].

The findings of Rafati et al. (2015) also showed that the level of empathy of medical students with patients varies during the academic years. In such a way that the level of empathy in medical students decreases with increasing age and educational level, which is in line with the findings of the present study. These researchers also believe that due to the importance of empathy as a moral virtue, it is necessary to plan to strengthen empathy and include this concept in the curriculum of medical students to improve the mental and spiritual health of patients [8]. Khairabadi et al.'s research also informs about the weakness in the field of empathy and the importance of its training [41]. Shariat and Kikhaoni (2009) in a study that measured the level of empathy in clinical assistants of Iranian medical sciences universities, reported the decrease of this skill in students during their studies [42]. Considering this shortcoming, although in order to reduce this dissatisfaction, empathy training workshops have been held for the treatment staff in some universities, but there is still a need for planning in this regard [43]. One of the important limitations of the present study is the involvement of intervening factors in the effect of medical students' empathy, which are uncontrollable. Another limitation of this study is the small number of female participants compared to male participants. This issue does not allow us to draw conclusions about gender differences. The next limitation was that giving the right to choose to participate in the research for students made only students who are interested in fiction to participate in it, and there is a possibility that these people are more prepared to teach empathy and be influenced by texts. Finally, considering the relationship between reading fiction and general empathy, it is suggested for future researches to examine the ranking of general empathy with real empathy and also how empathy research evolves over time.

## Conclusion

The findings of this research showed that story reading increased the general empathy of medical students in the fields of transfer of empathy, cognitive and emotional empathy in the post-intervention phase significantly more than the pre-intervention phase. This problem implicitly confirms the positive effect of reading stories in improving the sense of empathy. These results show the necessity of planning to include the study of stories and teaching them to read in the curriculum of medical students as a low-cost way to increase empathy. There is a literature course as a general course in the students' curriculum, but it does not have a specific educational framework. Considering the importance of storytelling in narrative medicine and the role of stories in creating empathy, it is suggested that more importance be given to reading stories in the curriculum of this course, especially during *Interns* and *Stagers*.

### Supplementary Information

**Additional file 1: Appendix 1.** The Stories.

## Data Availability

The datasets used and/or analysed during the current study are available from the corresponding author on reasonable request.

## References

[1] Samuel CA, Mbah O, Schaal J, Eng E, Black KZ, Baker S (2020). The role of patient-physician relationship on health-related quality of life and pain in cancer patients. Supportive Care Cancer.

[2] Halpern J (2007). Empathy and patient–physician conflicts. J Gen Intern Med.

[3] Yune SJ, Kang SH, Park K. Medical students' perceptions of patient-doctor relationship in South Korea: concept mapping analysis. Front Public Health. 2021:1606. 10.3389/fpubh.2021.658220 PMC8634939 34869134

[4] Van Der Merwe J (1995). Physician-patient communication using ancestral spirits to achieve holistic healing. Am J Obstet Gynecol.

[5] Rogers CR (1959). Significant learning in therapy and in education. Educ Leader.

[6] Khodabakhsh MR, Mansoori P (2011). Empathy and its impact on promoting physician-patients relationship. Iran J Med Ethics History Med.

[7] Kazemipoor M, Sattar BS, Hakimian R (2018). Patient empathy and related factors in undergraduate and postgraduate dental students.

[8] Shiva R, Nahid R, Ali D, Forotani F (2016). Empathic perspective of medical students based on Jefferson Empathy Scale. J Med Sci Res Ethics.

[9] Loftus S, Greenhalgh T. Towards a narrative mode of practice. Education for future practice: Brill; 2010. p. 85-94.

[10] Linda NC, Clement M (2023). The Application of Storytelling in Teaching and Learning: Implication on Pupil’s Performance and Enrolment in Schools.

[11] Cersosimo G (2019). Storytelling in medical education programs. Ital J Sociol Educ.

[12] Liao H-C, Wang Y-H (2020). Storytelling in medical education: narrative medicine as a resource for interdisciplinary collaboration. Int J Environ Res Public Health.

[13] Kamel-ElSayed S, Loftus S (2018). Using and combining learning theories in medical education. Med Sci Educ.

[14] Loftus S. The language of clinical reasoning. Clinical Reasoning in the Health Professions E-Book. 2018:129.

[15] Jones AH (2013). Why teach literature and medicine? Answers from three decades. J Med Humanit.

[16] Shapiro J, Nixon LL, Wear SE, Doukas DJ (2015). Medical professionalism: what the study of literature can contribute to the conversation. Philos Ethics Humanit Med.

[17] Sklar DP (2017). Health humanities and medical education: joined by a common purpose. Acad Med.

[18] Tamir DI, Bricker AB, Dodell-Feder D, Mitchell JP (2016). Reading fiction and reading minds: the role of simulation in the default network. Soc Cogn Affect Neurosci.

[19] Bonebakker V (2003). Literature & medicine: Humanities at the heart of health care: a hospital-based reading and discussion program developed by the Maine Humanities Council. Acad Med.

[20] Panahifar S, Nouriani JM (2021). The Effectiveness of Narrative therapy on Behavioral Maladaptation and Psychological Health of Children with ADHD in Kerman.

[21] Shahabizadeh F, Khageaminiyan F (2019). The effectiveness of narrative therapy based on cognitive-behavioral perspective on symptoms depression and dysthymic disorders in children. J Psychol Achieve.

[22] Azmi hjsa. farsi omomi, ahamiyat wa asibshenasi 1 th hamayesh amozeshe zabane farsi. 2016. p. 195-200.

[23] Kiyani barforoshi hR, ghodsiye. Pathological Criticism in farsi ye omomi; Critical Studies in Texts and Programs of Human Sciences 19. 2019;3:181-205.

[24] Boulter A. Writing fiction: creative and critical approaches: Bloomsbury Publishing; 2007.

[25] Damrosch D. How to read world literature: John Wiley & Sons; 2017.

[26] Foster TC (2014). How to Read Literature Like a Professor.

[27] Rasley A (2008). The Power of Point of View: Make Your Story Come to Life: Penguin.

[28] Robert S. Elements of Fiction: An Anthology. Oxford University Press; 1981.

[29] Truby J. The anatomy of story: 22 steps to becoming a master storyteller: Farrar, Straus and Giroux; 2008.

[30] Pino MC, Mazza M (2016). The use of “literary fiction” to promote mentalizing ability. PloS one.

[31] Bal PM, Veltkamp M (2013). How does fiction reading influence empathy? An experimental investigation on the role of emotional transportation. PloS one.

[32] Djikic M, Oatley K, Moldoveanu MC (2013). Reading other minds: effects of literature on empathy. Sci Study Lit.

[33] Mumper ML, Gerrig RJ (2017). Leisure reading and social cognition: a meta-analysis. Psychol Aesthetics Creativity Arts.

[34] Panero ME, Weisberg DS, Black J, Goldstein TR, Barnes JL, Brownell H (2016). Does reading a single passage of literary fiction really improve theory of mind? An attempt at replication. J Personal Soc Psychol.

[35] Dodell-Feder D, Lincoln SH, Coulson JP, Hooker CI (2013). Using fiction to assess mental state understanding: a new task for assessing theory of mind in adults. PloS one.

[36] Beaudoin C, Leblanc É, Gagner C, Beauchamp MH (2020). Systematic review and inventory of theory of mind measures for young children. Front Psychol.

[37] Kidd D, Ongis M, Castano E (2016). On literary fiction and its effects on theory of mind. Sci Study Lit.

[38] Koopman EME (2016). Effects of “literariness” on emotions and on empathy and reflection after reading. Psychol Aesthetics Creativity Arts.

[39] Engbretson AM, Poehlmann-Tynan JA, Zahn-Waxler CJ, Vigna AJ, Gerstein ED, Raison CL (2020). Effects of cognitively-based compassion training on parenting interactions and children’s empathy. Mindfulness.

[40] Stansfield J, Bunce L. The relationship between empathy and reading fiction: separate roles for cognitive and affective components. J Eur Psychol Students. 2014;5(3).

[41] Kheirabadi G H-RM, Mahki B, Masaiely N, Yahaei M, Golshani L,Kheirabadi D. Empathy with patients in medical sciences faculty physicians at Isfahan University of Medical Sciences, Iran. J Res Behav Sci. 2016;14(2):154-60.

[42] Shariat SV, Kaykhavoni A (2010). Empathy in medical residents at Iran University of Medical Sciences. Iran J Psychiatry Clin Psychol.

[43] Managheb E, Bagheri S (2013). The impact of empathy training workshops on empathic practice of family physicians of Jahrom University of Medical Sciences. Iran J Med Educ.

