# Supplementary material for: Designing, implementation and evaluation of story reading: a solution to increase general empathy in medical students
Source: BMC Med Educ. 2024 Apr 30;24:477. doi: 10.1186/s12909-024-05384-4 (PMC11061996; doi:10.1186/s12909-024-05384-4)
Supplement: Supplementary file 1 — Additional file 1: Appendix 1. The Stories. [file 12909_2024_5384_MOESM1_ESM.docx]

Appendix 1: The Stories

| The first session: " Arosak- e- Foroshi” (1965), Sadegh Chubak, collection of stories “Roze Avale Ghabr”, Scientific Publications (from the collection of short stories in Iran); Teaching the story elements of the topic, theme and plot in realistic stories |
| --- |
| The second session: “Maraze Heyvan” (2007), Peyman Esmaili, Collection of stories “Barf va Samfoniye Abr”, Cheshme publication, winner of the 10th edition of the Press Critics Award 2008, winner of the the best story collection from Rozi Rozgari Literary Award, Golshiri Foundation Award, and Mehrgan Award 2007; Teaching the elements of conversation and scene (story world), narration and multiple narrators |
| The third session: “Mordeghan” (2004), Mohammad Hossein Mohammadi, collection of stories “Anjirhaye Sorkh Mazar”, published by Cheshme, winner of the Golshiri Foundation prize in 2004; Familiarity with modern story and the element of perspective and its importance to understand the concept |
| The fourth session: “Seporde be Zamin” (1994), Bijan Najdi, collection of stories “Uzpalangani ke ba Man Davideand“, published by the Center, winner of the Golden Pen Award of the Gerdon Award 1995 (festival selection); Getting to know symbolic language in the modern lyrical story |
| The fifth session: “Yekbar Ham Shode Sosun Ghosh Bede” (2007), Peyman Houshmandzadeh, collection of stories "Ha Kardan", third edition, Cheshme Publishing House; Getting to know the element of character and characterization in modern fiction |
| The sixth session: “Mizgerd“ (1997) by Simin Daneshvar, collection of “Az Parandegane Mohajer Bepors“ ,Kanoon New Publications (from the collection of short stories in Iran); Familiarity with the postmodern story, Plot and its components |
